# Impact of iron ore mining activities on the rhizosphere fungal communities of *Musa basjoo*, *Apegdalus persica*, and *Triticum aestivum* L.

**DOI:** 10.3389/fmicb.2025.1592479

**Published:** 2025-06-27

**Authors:** Caijing Wan, Xinhui Wang, Yuan Luo, Sumin Chen, Shuyi Chen, Xin Yu, Ying Wang, Bingliang Liu, Qiang Li

**Affiliations:** ^1^School of Food and Biological Engineering, Chengdu University, Chengdu, Sichuan, China; ^2^Chengdu Agricultural Science and Technology Center, Chengdu, Sichuan, China

**Keywords:** iron ore, soil fungus, high-throughput sequencing, FunGuild, rhizosphere soil

## Abstract

Iron ore mining has damaged the natural environment and affected the ecological balance of the surrounding areas. The purpose of this study was to investigate the effects of iron ore mining on fungal communities in the rhizosphere soils of plantain, peach, and wheat. The results revealed that, due to iron ore mining, the pH value of the soil in the mining area generally exhibited weak acidity, and the Fe and TP contents increased significantly (*P* < 0.05). Notably, iron ore mining reduced fungal diversity in the rhizosphere, and only the wheat group presented a significant reduction in fungal diversity (*P* < 0.05). Moreover, the relative abundances of *Cladosporiaceae*, *Cladosporium*, and *Sarocladium* in the soil samples decreased due to iron ore mining. Conversely, the relative abundances of *Fusarium* and *Mortierella* in the soil samples were markedly enhanced by mining activities (*P* < 0.05). Beta diversity analysis revealed significant differences between the rhizosphere soil fungal communities of the three plants growing in the iron ore area and those growing in the non-iron ore area. The degree of variation among the different plants also varied. The Animal Pathogen-Endophyte-Fungal Parasite-Plant Pathogen-Wood Saprotroph guild was the dominant guild in the rhizosphere soil of the plants in the mining area. In summary, the results of this study help elucidate the effects of iron ore mining on fungal communities in the rhizosphere soil and provide a theoretical basis for screening fungi that can restore the ecology of the iron ore mining area.

## 1 Introduction

Mining activities have a long history, and humans have obtained corresponding mineral resources through mining activities. As an important support for national development, mineral resources have greatly promoted socioeconomic development and national industrialization. However, these large-scale mining activities not only cause damage to the natural environment but also may have a profound impact on the surrounding ecological balance (Sun et al., 2024; Vinayagam et al., 2024). These impacts can still be present even decades after mining has ceased (Diami et al., 2016). In recent years, environmental problems caused by heavy metal pollution have become increasingly significant. The presence of heavy metals in the environment can be traced back to mining activities (Li et al., 2014; Martínez López et al., 2008; Ding et al., 2017).Additionally, research has indicated that the spatial arrangements of soil and microorganisms, along with the ways in which microbes interact, vary between mining and non-mining regions (Chen et al., 2020a; Rime et al., 2015). Yunnan, positioned at the junction of the Eurasian Plate and the Indian Plate, possesses a complex geological structure, optimal ore-forming conditions, and an incredibly vast array of mineral resources (Hillman et al., 2015), known as the “Mineral Kingdom.” A risk assessment of comprehensive environmental and biological factors was conducted for heavy metals in the soil of specific sources of arsenic waste mining areas in Yunnan Province (Huang et al., 2025). As, Cd, and Pb were found to be the primary pollutants causing ecological health risks, with mining and smelting pinpointed as the key sources for health risk control (Huang et al., 2024). In addition, mining and smelting activities generate large amounts of atmospheric dust, wastewater, and tailings, which also cause severe heavy metal pollution in surrounding areas (Guo et al., 2019).

In an ecosystem, the rhizosphere of a plant is an energetic and dynamic interface that contains many microbial communities. Within the rhizosphere community, the fungal community plays a crucial role and can be classified into symbiotic fungi, saprophytic fungi, and endophytic fungi or pathogenic fungi (Gaggini et al., 2018). Fungi are widely spread in soil and are crucial for both soil nutrient conversion and the breakdown of plant litter (Nagati et al., 2018; Zeilinger et al., 2016). In general, different environmental factors cause different ecological succession processes, while the development of fungal communities under different environmental factors can vary unpredictably (Kolaříková et al., 2017). The composition and structure of the rhizosphere fungal community, however, are influenced by local biotic and abiotic conditions (Alzarhani et al., 2019). Human activities, such as mining, may generate pollutants such as dust and wastewater, which can enter the soil (Park et al., 2015), changing the physicochemical properties of the soil can affect the survival and reproduction of rhizosphere fungi. In addition, mining activities may destroy the soil structure (Xie et al., 2023; Wang et al., 2024), leading to soil erosion and nutrient loss, further affecting crop growth and the stability of the rhizosphere fungal community.

*Musa basjoo* (Japanese banana), a member of the *Musaceae* family, is a favorite fruit in tropical and subtropical areas and has certain medicinal value (Wei et al., 2019; Matsumoto et al., 2022). In Yunnan, plantain planting not only provides local residents with rich fruit resources but also drives the development of related industries, such as the production of processed products such as dried plantains and plantain powder. *Apegdalus persica* (peach) is grown in a wide range of temperate and subtropical climate zones worldwide and is a popular fruit (Noor et al., 2023). Moreover, peaches not only taste delicious but also contain a variety of nutrients that are beneficial to the human body, including polyphenols, soluble sugars, organic acids, vitamins, and rich minerals (Kim et al., 2019; Qi et al., 2024). *Triticum aestivum* L. (common wheat) is considered one of the most significant food crops in human history (Gupta et al., 2020), is rich in many minerals, vitamins, and phytochemicals and has high nutritional value (Zörb et al., 2006; Hawkesford, 2017). Soil fertility plays a crucial role in determining the yield of wheat. Fertile soil can provide the nutrients and water needed for wheat growth and promote the growth and development of wheat (Chen et al., 2023; He et al., 2024; Liu Y. et al., 2024). In general, *Musa basjoo*, *Apegdalus persica*, and *Triticum aestivum* L. are widely planted in Yunnan Province. Each is representative and has an important influence on agricultural production and economic development in Yunnan Province.

Soil microbes’ abundance and community structure are greatly influenced by environmental pollution (Shuaib et al., 2021; Zhou et al., 2024). The growth and enrichment of certain microorganisms with efficient tolerance and degradation abilities in environmentally contaminated soil lead to the improvement of the soil environment through their physiological activities. This provides benefits for plants growing in contaminated areas and creates conditions conducive to their thriving (Das et al., 2017). Hence, it is crucial to conduct a thorough examination of how environmental pollution impacts the structure and function of soil microbial communities in order to pinpoint and evaluate microbial species that have the ability to effectively remediate environmental pollution. In Yunnan mining regions, certain studies have specifically examined heavy metal contamination in soils (Cheng et al., 2018; Liu et al., 2022; Yanqun et al., 2005). Nonetheless, there is a scarcity of research on how iron ore mining activities affect fungal communities in rhizosphere soils.

In this study, to address the potential threat of iron ore mining to plant rhizosphere fungi, the rhizosphere soils of three dominant plants in the mining area were selected to investigate the mechanism of mining disturbance on their microbial diversity and stability. Through a detailed examination of the physicochemical properties of rhizosphere soil and the composition and diversity of rhizosphere fungal communities in both the iron ore area and the non-iron ore area, we could elucidate the specific impact of iron ore mining activities on rhizosphere soil fungal communities. Additionally, this research can lay the groundwork for identifying fungi that may aid in the restoration of the ecological balance in iron ore mining regions.

## 2 Materials and methodology

### 2.1 Rhizosphere soil sample collection

The iron ore is located in Wuding County, Chuxiong Yi Autonomous Prefecture, Yunnan Province (102.40° east longitude, 25.53° north latitude). The climate in this area is characterized by a plateau monsoon climate at low latitudes, showcasing large daily temperature variances, slight annual temperature changes, irregular precipitation with abundant summers and autumns, significant vertical climate discrepancies, and prolonged periods of sunshine. By observing the planting conditions and growth status of local plants, we selected the rhizosphere soils of three plants, *Musa basjoo* (Japanese banana), *Apegdalus persica* (peach) and *Triticum aestivum* L. (common wheat), as the research objects. In April 2024, the rhizosphere soils of three plants in the iron ore area were collected, namely, *Musa basjoo* (sample No. Mba), *Apegdalus persica* (sample No. Ape), and *Triticum aestivum* L. (sample No. Tae). Rhizosphere soil and blank soil samples were subsequently collected from non-mining areas under similar environmental conditions: *Musa basjoo* (Sample No. CK-Mba), *Apegdalus persica* (Sample No. CK-Ape), *Triticum aestivum* L. (Sample No. CK-Tae) and CK. Three 1 m × 1 m quadrats were randomly set up for each plant. One soil sample was obtained by evenly mixing multiple points in the same quadrant. Three parallel samples were collected from each group and 50 g of rhizosphere soil was collected from each sample, totaling 21 samples. After the soil layer from the surface to a depth of 5 cm was removed, the root system of the plants and the accompanying soil samples were systematically sampled for analysis. The loose soil was removed by shaking the roots, and then 0–5 mm of soil was collected from the roots with a sterile brush as rhizosphere soil. The operator wore sterile gloves throughout the process to prevent any potential contamination of the samples. The collected soil samples were divided into two parts for processing: one part was immediately loaded into sterile sampling bags and stored at −80°C in a low-temperature environment for subsequent soil microbial DNA extraction and high-throughput sequencing analysis. The other part was used to evaluate the soil physicochemical properties and detect heavy metal contents.

### 2.2 Soil physicochemical properties and heavy metal detection

The pH value was measured using a pHS-3 pH meter (Shanghai Yidian Scientific Instrument Co., Ltd., China) after being mixed evenly at a ratio of soil to water of 1:2.5 (Ren et al., 2022). The soil organic matter (OM) content was evaluated by the potassium dichromate oxidation method (Kalembasa and Jenkinson, 1973). The total nitrogen (TN) content was determined using the concentrated sulfuric acid digestion-Kjeldahl method (Bremner, 1960). The total phosphorus (TP) content was measured using the antimony anti-colorimetric method (Sommers and Nelson, 1972). The total potassium (TK) content was evaluated by the sodium carbonate melting method (Knudsen et al., 1982). The alkaline nitrogen (AN) content was determined using the Mason jar diffusion method (Bremner, 1996). Available phosphorus (AP) was determined using the molybdenum blue colorimetric method (Tan et al., 2014). Available potassium (AK) was extracted via NH_4_OAC and flame photometry (Zhao et al., 2023). The contents of heavy metals [iron (Fe), titanium (Ti), copper (Cu), zinc (Zn), and lead (Pb)] were determined via ICP-AES (Icap 6000, Thermo Fisher, United Kingdom) (Mosallaei et al., 2023). Each soil sample was measured at least three times, and the mean value was calculated.

### 2.3 Genomic DNA extraction and PCR amplification

Genomic DNA was extracted using the CTAB method (Wang H. et al., 2023). The purity and concentration of the extracted DNA were examined by agarose gel electrophoresis, and the DNA was diluted with sterile water to 1 ng/μL. The ITS1 region of these samples was amplified via the specific primers ITS5-1737F (5′-GGAAGTAAUGUGTCGTAACAAGG-3′) and ITS2-2043R (5′-GCTGCGTCTTCATCGATGC-3′) with specific barcodes. Using the diluted genomic DNA as a template, PCR was performed according to the amplified region using specific primers with barcodes, New England Biolabs’ Phusion^®^ High-Fidelity PCR Master Mix with GC Buffer, and high-efficiency high-fidelity enzymes to ensure efficient and accurate amplification. First, initial denaturation at 95°C for 3 min was performed, followed by sequential amplification cycles of denaturation at 95°C, annealing at 50°C for 30 s, and extension at 72°C for 30 s. Finally, another extension was performed at 72°C for 5 min. Then, the PCR products were analyzed and purified by 2% agarose gel electrophoresis.

### 2.4 Library construction, computer sequencing, and raw data processing

We constructed sequencing libraries using the TruSeq^®^ DNA PCR-Free Sample Preparation Kit (Illumina, CA, United States) following the manufacturer’s instructions and adding index codes. The library quality was subsequently comprehensively evaluated using a Qubit^®^ 2.0 fluorescence analyzer (Thermo Scientific, MA, United States) and an Agilent Bioanalyzer 2100 system (Agilent, Beijing, China). Finally, we successfully sequenced the library on the Illumina NovaSeq sequencing platform and obtained 250-bp paired-end reads. Next, based on the unique barcode of each sample, we accurately assigned the generated paired-end reads to the corresponding sample and truncated them by removing the barcode and primer sequences. After that, we used FLASH software (Magoč and Salzberg, 2011) to merge the data from the paired reads into the original tag. For quality control, we followed the quality control process of QIIME v1.9.1 and conducted strict quality screening of the original markers under specific filtering standards to obtain high-quality clean markers. To detect and remove chimeric sequences, we aligned the markers to the reference Silva database (Haas et al., 2011). Ultimately, effective markers for downstream analysis were obtained by aligning them to the reference Silva database.

### 2.5 ASV noise reduction and species annotation

For the effective markers obtained above, the DADA2 software in QIIME2 (Callahan et al., 2016) was used to reduce noise and filter out sequences with an abundance of less than 5 (Li et al., 2020) to obtain the final ASVs (Callahan et al., 2017) (amplicon sequence variants, i.e., amplicon sequence variation) and feature table. The classify-sklearn function in QIIME2 software was subsequently used (Bokulich et al., 2018; Bolyen et al., 2019). The module compares the obtained ASVs with the database to obtain the species information of each ASV.

### 2.6 Alpha diversity and species composition analysis

Alpha diversity was used to assess microbial community richness and evenness (Li et al., 2013), which can reflect the richness and diversity of the microbial communities in a sample. The QIIME2 software was used to calculate the observed OTUs, Shannon, Simpson, Chao1, dominance, and Pielou evenness indices. Additionally, the rarefaction curve and species accumulation boxplot were generated. On the basis of the ASV annotation results and the characteristics of each sample, species abundance tables were obtained at the class, order, family, and genus levels. These tables were then analyzed in combination with the species composition results of different sample groups.

### 2.7 Beta diversity

Beta diversity is a comparative analysis of the microbial community composition of different samples. First, based on the species annotation results and the abundance information of the ASVs for all samples, the information of ASVs with the same classification was merged and processed to obtain an abundance profiling table for species. Moreover, weighted Unifrac distances (Unifrac) were calculated using QIIME2 software using phylogenetic relationships among ASVs (Lozupone and Knight, 2005; Lozupone et al., 2011). The Unifrac distance is a method that uses evolutionary information between microbial sequences in each sample to calculate the intersample distance. If there are more than two samples, a distance matrix is obtained. The weighted Unifrac distance was subsequently constructed from the weighted Unifrac distance using the abundance information of the ASVs (Lozupone et al., 2007). Finally, intergroup difference analysis of the beta diversity index, principal coordinate analysis (PCoA), and the non-metric multidimensional scaling (NMDS) method was used to identify differences among different sample groups.

### 2.8 Functional prediction and correlation analysis of the fungal community

Through ITS amplicon analysis, FunGuild tool (V 1.1) can identify the types and abundances of fungi in the environment. More importantly, it can also classify fungi according to their species, revealing the specific functions of these fungi in the ecosystem. Based on the database annotation results, the functional abundance results were counted in guild and trophic modes. Based on the functional annotation and abundance information of the samples in the database, the top 35 most abundant functions and their abundance information were selected to draw heatmaps for each sample. Clustering was then performed at the level of functional differences.

### 2.9 Statistical analysis

All variables (e.g., alpha diversity indices, fungal abundances) were tested for normality using the Shapiro-Wilk test (*P* > 0.05) and visually inspected using Q-Q plots. For non-normally distributed data, non-parametric tests were applied. Homogeneity of variances was verified using Levene’s test. For data satisfying normality and homogeneity of variances, a one-way ANOVA was performed. If ANOVA revealed significant differences (*P* < 0.05), the Tukey’s HSD *post-hoc* test was used for pairwise comparisons. For non-parametric data, the Kruskal-Walli’s test was conducted, followed by Dunn-Bonferroni correction for multiple comparisons. Statistical significance was set at *P* < 0.05.

## 3 Results and analysis

### 3.1 Basic physicochemical properties and heavy metal contents of rhizosphere soil

Table 1 displays the fundamental physicochemical characteristics and heavy metal concentrations of the rhizosphere soils in both the iron ore area and non-iron ore area. We measured the pH, organic matter (OM), total nitrogen (TN), total phosphorus (TP), and total potassium (TK), alkaline nitrogen (AN), available phosphorus (AP), and available potassium (AK). The rhizosphere soils of the three plants grown in the mining area exhibited a significant increase (*P* < 0.05) in organic matter (OM) and total potassium (TK) content compared to those grown in the non-mining area. The presence of this phenomenon indicates two potential scenarios: the soil in the iron ore mine region is naturally abundant in organic matter and potassium, or the mining operations have hastened the introduction and buildup of organic matter and potassium in the soil. Soils in the iron ore region were commonly acidic. The rhizosphere soil of CK-Mba was strongly acidic, while the acidity of the Mba rhizosphere soil decreased and was acidic. However, the differences between Ape and CK-Ape and between Tae and CK-Tae were more significant. The rhizosphere soil of CK-Ape was alkaline, but the rhizosphere soil of Ape was acidic. The rhizosphere soil of CK-Tae was weakly alkaline, whereas the rhizosphere soil of Tae was weakly acidic. Compared with those in CK-Mba, the contents of TN and AN in the rhizosphere soil of Mba significantly increased (*P* < 0.05). Compared with those of CK-Ape and CK-Tae, the contents of TN and AN in the root soils of Ape and Tae significantly increased compared with those of CK-Ape and CK-Tae, respectively. The AN content significantly decreased (*P* < 0.05), and the AN content in the CK-Mba rhizosphere soil was the lowest among all the samples. In terms of phosphorus, the changes in phosphorus content were different from those in nitrogen content. Compared with that of CK-Mba, the TP content significantly increased (*P* < 0.05), whereas the AP content significantly decreased (*P* < 0.05). In contrast, compared to those in CK-Ape and CK-Tae, the AP content in the root soil of Ape and Tae significantly decreased (*P* < 0.05), while the TP content significantly increased (*P* < 0.05). Compared with the rhizosphere soil of the non-iron ore area, the AK content of the Mba and Tae rhizosphere soils growing in the mining area was significantly greater (*P* < 0.05), while the AK content of the Ape rhizosphere soil was significantly lower (*P* < 0.05).

**TABLE 1:** Basic physical and chemical properties of plant rhizosphere soils.

	Mba	CK-Mba	Ape	CK-Ape	Tae	CK-Tae	CK
pH	5.67 ± 0.0	5.30 ± 0.0	5.79 ± 0.0	7.55 ± 0.0	6.01 ± 0.0	7.42 ± 0.0	6.36 ± 0.0
	3f	2g	1e	2a	1d	2b	4c
Organic matter (g⋅kg^–1^)	76.90 ± 2.	24.62 ± 0.	137.45 ± 2	115.27 ± 1	88.89 ± 1.	79.72 ± 0.	42.61 ± 0.
	36e	33g	0.45a	0.27b	36c	66d	40f
Total nitrogen (g⋅kg-’)	2.39 ± 0.0	1.48 ± 0.0	3.25 ± 0.0	5.67 ± 0.0	3.14 ± 0.0	3.53 ± 0.0	1.95 ± 0.0
	1a	1b	2c	1d	2e	4f	2g
Total phosphorus (g⋅kg^–1^)	0.91 ± 0.0	0.77 ± 0.0	0.94 ± 0.0	4.94 ± 0.0	1.13 ± 0.0	1.63 ± 0.0	1.03 ± 0.0
	1e	4f	1e	3a	1e	3b	1d
Total potassium (g⋅kg^–1^)	20.20 ± 0.	5.94 ± 0.1	20.81 ± 0.	9.79 ± 0.1	23.08 ± 0.	14.77 ± 0.	0.98 ± 0.0
	15c	8f	18b	6e	21a	17d	2g
Avaliable nitrogen (mg⋅kg^–1^)	394.19 ± 2	172.53 ± 1	553.13 ± 3	746.04 ± 2	353.64 ± 1	439.99 ± 3	193.09 ± 2
	0.09d	0.94g	0.42b	0.13a	0.67e	0.47c	0.64f
Available phosphorus (mg⋅kg^–1^)	14.37 ± 0.	21.71 ± 1.	28.48 ± 0.	18.60 ± 0.	70.74 ± 0.	18.10 ± 0.	66.52 ± 0.
	79f	32d	20c	66e	87a	99e	62b
Avaliable potassium (mg⋅kg^–1^)	387.60 ± 3	331.10 ± 3	336.60 ± 3	838.69 ± 8	588.52 ± 4	526.59 ± 4	256.41 ± 2
	0.88d	0.04e	0.29e	0.34a	0.56b	0.44c	0.33f
Fe/(g⋅kg^–1^)	72.55 ± 3.	48.18 ± 1.	68.64 ± 2.	42.43 ± 1.	70.54 ± 2.	56.04 ± 1.	43.54 ± 1.
	69a	16c	52a	54d	31a	61b	35d
Ti/(g⋅kg^–1^)	4.75 ± 0.2	5.23 ± 0.1	3.92 ± 0.1	6.21 ± 0.2	4.40 ± 0.2	5.19 ± 0.1	4.80 ± 0.2
	6c	8b	1d	6a	1e	9b	1e
Zn/(mg⋅kg^–1^)	55.15 ± 2.	29.33 ± 1.	63.45 ± 2.	215.41 ± 1	48.37 ± 1.	82.20 ± 2.	53.54 ± 2.
	44d	07e	10c	0.06a	20d	76b	34d
Cu/(mg ⋅kg^–1^)	52.81 ± 2.	21.14 ± 0.	65.11 ± 0.	184.36 ± 9	20.18 ± 1.	69.04 ± 3.	45.15 ± 1.
	21c	88e	98b	0.38a	01e	44b	30d
Pb/(mg⋅kg^–1^)	50.01 ± 1.	40.23 ± 1.	47.29 ± 2.	29.20 ± 1.	33.20 ± 1.	49.14 ± 2.	39.35 ± 1.
	73a	72b	14a	52d	01e	07a	84b

Apart from basic physicochemical properties, several heavy metal elements were also detected in the seven samples, as indicated in Table 1. There was no significant change in the Fe content in the rhizosphere soil among the three plants grown in the iron ore mine area, whereas it showed a significant increase (*P* < 0.05) compared to the three plants in the non-iron ore mine area. However, the Ti content in the rhizosphere soil of the three plants that were planted in the mine area decreased significantly (*P* < 0.05) as opposed to the three plants grown in the non-iron ore area. The Cu and Zn contents of Mba rhizosphere soil significantly increased compared to CK-Mba, but decreased significantly in Ape and Tae root soils compared to CK-Ape and CK-Tae (*P* < 0.05). In the mining area, the Pb contents of Mba and Ape were significantly greater (*P* < 0.05) than those in the non-mining area, whereas the Pb content in the rhizosphere soil of Tae was significantly lower (*P* < 0.05) than that in CK-Tae.

In general, the mining environment has a certain effect on the physicochemical properties of plant rhizosphere soil. The rhizosphere soil of plants in the iron ore area was slightly acidic, with higher levels of OM, TK, and Fe compared to the control groups (CK-Mba, CK-Ape, and CK-Tae) (*P* < 0.05). However, the Ti content in the rhizosphere soil of Mba, Ape, and Tae was markedly above that in the rhizosphere soil of CK-Mba, CK-Ape, and CK-Tae, respectively (*P* < 0.05). Moreover, the TK and Fe content in the rhizosphere soil of the three plants in the iron ore region were typically higher compared to those in the rhizosphere soil in the non-iron ore region.

### 3.2 Alpha diversity analysis

Sparse curves were used to assess whether the sequencing depth was sufficient to cover the diversity of the community and to compare diversity differences between samples. As shown in Supplementary Figures 1, 2, when the curves flatten out, it indicates that the current amount of sequencing data is saturated and that continuing to add data has little effect on the estimation of the alpha diversity index.

**FIGURE 1:**
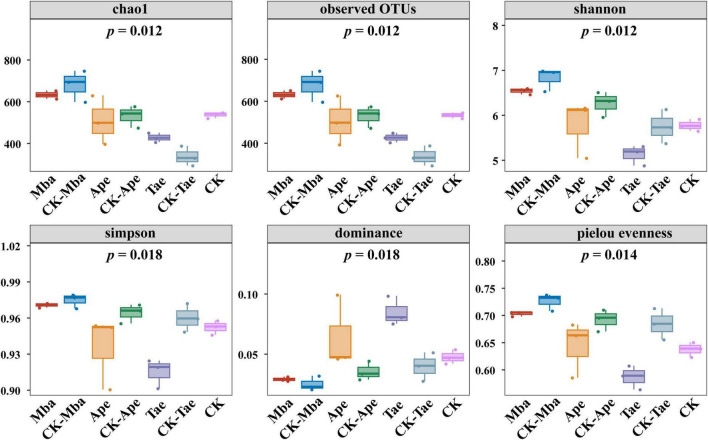
Box plots of fungal diversity and richness in different samples.

Alpha diversity was used to assess within-community microbial diversity. Alpha Diversity analysis indices (observed OTUs, shannon, simpson, chao1, goods_coverage, dominance, and Pielou evenness) were calculated for various samples using QIIME2 software and displayed on a Species Accumulation Boxplot, as depicted in Figure 1. The analysis of community richness incorporated the Chao1 and observed OTUs indices, with a higher index indicating a larger number of species. The Chao1 and observed OTUs indices of the rhizosphere soil in Mba and Ape showed a decrease in comparison to those in the non-iron ore area, whereas the Chao1 and observed OTUs indices of the rhizosphere soil in Tae displayed an increase. However, these changes were not significant. The diversity of a community can be measured using the Shannon and Simpson indices, with a higher index indicating a more diverse range of species. In this study, the values of the three types of rhizosphere soils in the iron ore area were generally lower than those in the non-iron ore area, indicating that iron ore mining activities reduced the diversity of fungal communities in the rhizosphere soil. The Pielou evenness is an evenness index. The more uniform the species is, the larger the Pielou evenness is. The Pielou evenness of the rhizosphere soils of the three plants grown in the mining area in this study were lower than those of plants from the non-mining area, suggesting that iron ore mining reduced the evenness of the fungal community in the plant rhizosphere soil.

### 3.3 Analysis of the composition of the fungal community in rhizosphere soil

**FIGURE 2:**
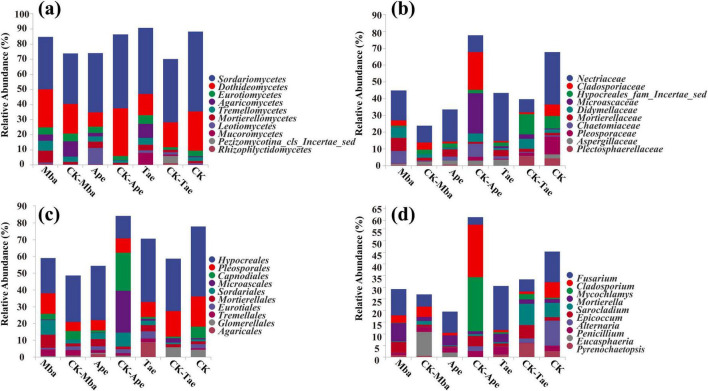
Relative abundance of plant rhizosphere soil fungi at the order level **(a)**, phylum level **(b)**, family level **(c),** and genus level **(d)**.

We counted the reads using QIIME2 (2019.4). We visually displayed the distribution of composition for each sample at the four classification levels: class, order, family, and genus. We further examined the individual species composition of every sample. In Figure 2a, we compared the ten most abundant classes across different samples at the class level. Among all the samples, the fungal species with the highest content were *Sordariomycetes*, *Dothideomycetes*, *Eurotiomycetes*, *Agaricomycetes*, and *Tremellomycetes*, which accounted for 42.10% ± 1.82%, 20.38% ± 0.20%, 3.93% ± 0.23%, 3.83% ± 2.13%, and 3.21% ± 0.45%, respectively, of the rhizosphere fungi in all samples. Compared with that in the non-iron ore area, the abundance *of Sordariomycetes* in the rhizosphere soil of Mba and Tae in the iron ore area increased, whereas the abundance *of Sordariomycetes* in the Ape rhizosphere soil decreased significantly (*P* < 0.05). Compared to those in the non-iron ore area, the abundances of *Sordariomycetes*, *Mortierellomycetes*, and *Leotiomycetes* in the rhizosphere soil of the three plants in the iron ore area increased. This indicates that mining activities may affect fungal diversity in plants.

In the category of orders, we compared the 10 orders with the highest abundance in different samples. In Figure 2b, the most abundant fungi in all samples were *Hypocreales, Pleosporales*, *Capnodiales*, *Microascales*, and *Sordariales*, making up 29.32% ± 1.58%, 10.66% ± 0.70%, 5.93% ± 0.58%, 4.25% ± 0.68%, and 4.00% ± 0.29% of the rhizosphere fungi, respectively. Compared with that in non-iron ore areas, the abundance of *Hypocreales* in the rhizosphere soil of Mba in the iron ore area decreased significantly (*P* < 0.05), whereas the abundance *of Hypocreales* in the Ape and Tae rhizosphere soils increased significantly (*P* < 0.05). In the iron ore area, the abundance of *Pleosporales* in the rhizosphere of Mba significantly decreased (*P* < 0.05), while the abundance of *Pleosporales* in the rhizosphere of Ape increased. Additionally, the abundance of *Pleosporales* in the rhizosphere of Tae significantly increased, and the abundance of *Pleosporales* in the Tae rhizosphere also increased. The abundance of *Mortierellales* and *Tremellales* in the rhizosphere soil of the three plants in the iron ore area was greater than that in the non-iron ore area. Among them, the abundance *of Mortierellales* in the rhizosphere soils of Mba and Ape increased significantly, and the abundance *of Tremellales* in the Tae rhizosphere soil increased significantly.

In the category of families, we compared the 10 families with the highest abundance in different samples. In Figure 2c, it can be observed that the most common fungi present in all samples were *Nectriaceae*, *Cladosporiaceae, Hypocreales_fam_Incertae_sedis*, *Microascaceae*, and *Didymellaceae*, which collectively represented 17.85% ± 0.28%, 5.72% ± 0.56%, 4.26% ± 0.59%, 4.07% ± 0.63%, and 3.37% ± 0.03% of the rhizosphere fungi in all samples. Compared with those in the rhizosphere soil of the plants growing in the non-iron ore area, the abundance of *Nectriaceae* in the rhizosphere soil of the three plants growing in the iron ore area significantly increased (*P* < 0.05), whereas the abundances of *Cladosporiaceae* and *Pleosporaceae* decreased. Only the abundance of *Cladosporiaceae* in the rhizosphere soil of Ape has significantly decreased (*P* < 0.05). Compared with that in the rhizosphere soil of the non-iron ore area, the abundance of *Mortierllaceae* in the rhizosphere soil of the three plants in the iron ore area increased, and only the Mba and Ape groups significantly changed (*P* < 0.05).

In the category of genus, we compared the 10 genus with the highest abundance in different samples. In Figure 2d, the most abundant fungi in all samples were *Fusarium*, *Cladosporium*, *Mycochlamys*, *Mortierella*, and *Sarocladium*, making up 9.47% ± 0.19%, 5.70% ± 0.56%, 3.86% ± 0.59%, 3.13% ± 0.60%, and 2.74% ± 0.35% of the rhizosphere fungi, respectively. The abundance of *Fusarium* in the rhizosphere soil of plants growing in the iron ore area was significantly greater compared to those in the non-iron ore area (*P* < 0.05). Additionally, the abundance of *Mortierella* in the rhizosphere soil of the three plants growing in the iron ore area showed an increase compared to those in the CK group. However, the abundance of *Mortierella* in the Mba and Ape rhizosphere soils significantly increased (*P* < 0.05) compared to the CK group. Moreover, compared with those in the rhizosphere soil of the iron ore area, the abundances of the three plant species growing in the rhizosphere soil in the iron ore area decreased: *Cladosporium*, *Sarocladium*, and *Alternaria*. Among them, the abundance of *Cladosporium* in the Ape rhizosphere soil and the abundance of *Sarocladium* in the Tae rhizosphere soil decreased significantly (*P* < 0.05). Compared with that in the rhizosphere soil in the non-iron ore area, the abundance of *Mycochlamys* in the Mba rhizosphere soil in the iron ore area increased, while the *Mycochlamys* abundance in the Ape and Tae rhizosphere soils decreased. Only the Ape rhizosphere soil exhibited a significant change in *Mycochlamys* abundance (*P* < 0.05).

**FIGURE 3:**
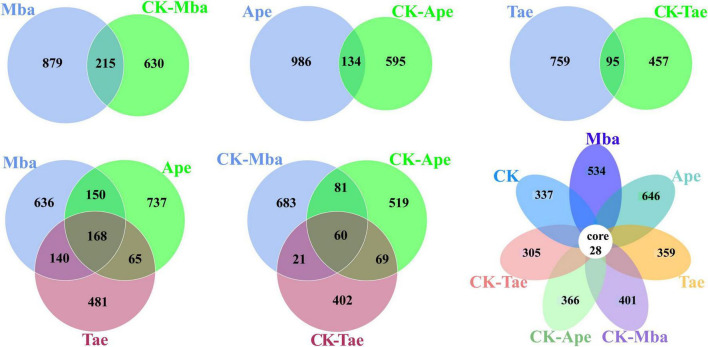
Shared and unique OTUs between different samples.

For the purpose of visualizing the difference in species composition between the samples and understanding the structural differentiation of the soil fungal community, an ASV Venn diagram was created to pinpoint the unique and common ASVs among the samples. The results are displayed in Figure 3. There were a total of 215 ASVs between the Mba and CK-Mba samples, and the number of specific ASVs was 879 and 630, respectively. There were 134 ASVs between the Ape and CK-Ape samples, with 986 and 595 specific ASVs, respectively. There were a total of 95 ASVs between the Tae and CK-Tae samples, with 759 and 457 specific ASVs, respectively. There were 60 ASVs in the rhizosphere soil samples of the three plants planted in the iron ore area, and the specific ASVs for Mba, Ape and Tae were 683, 519, and 402, respectively. There were 168 ASVs in the rhizosphere soil samples of the three types of plants planted in the non-iron ore area, and there were 636, 737 and 481 ASVs specific to CK-Mba, CK-Ape and CK-Tae, respectively. There were 28 ASVs among all the samples. In addition, 534, 646, 359, 401, 366, 305, and 337 ASVs were specific to the Mba, Ape, Tae, CK-Mba, CK-Ape and CK-Tae samples, respectively. There was a notable increase in the number of ASVs in the rhizosphere soil samples of plants growing in the iron ore area, as opposed to those in the non-iron ore area, indicating a disturbance in fungal communities due to iron ore mining.

### 3.4 Structural differentiation of soil fungal communities

**FIGURE 4:**
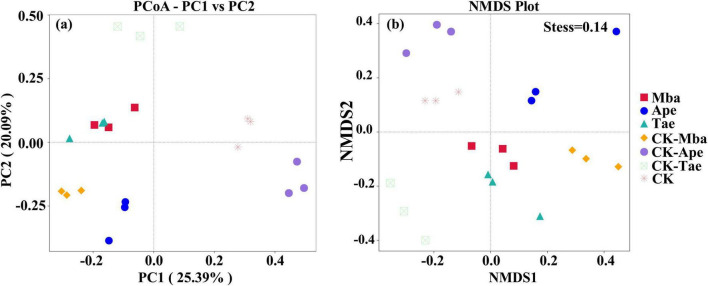
Beta diversity analysis of fungal communities in different samples based on PCoA **(a)** and NMDS **(b)** analysis.

We used a fungal PCoA analysis based on the Bray-Curtis dissimilarity distance matrix to compare differences in community structure between samples. The rate of explanation of differences in sample composition by each principal coordinate component of the PCoA analysis is represented by percentages, while points of different colors or traits represent samples of different groupings. The closer the distance between the sample points, the more similar the species composition of the two samples. As shown in Figure 4a, the PC1 principal axis explained 25.39% and the PC2 principal axis explained 20.09%, which cumulatively explained 45.48% of the variance in sample composition. Moreover, it is obvious from the figure that there is a certain distance between the sample points of the three plants grown in the mining area and the sample points of the three plants grown in the non-mining area. This indicates that there is a difference in the structure of the rhizosphere soil fungal communities of the three plants grown in the mining area and those grown in the non-mining area. The NMDS analysis was also performed based on the Weighted_unifrac matrix, and the Stress value was < 0.2, indicating that the NMDS analysis results were more reliable. As shown in Figure 4b, for the same plant, the distance between samples was closer in the same environment and farther between samples in different environments. This indicates that the structure of the rhizosphere fungal community composition of the same species differed more between mining and non-mining environments. Overall, the PCoA analysis yielded consistent results with the NMDS analysis.

### 3.5 Community function prediction and difference analysis

**FIGURE 5:**
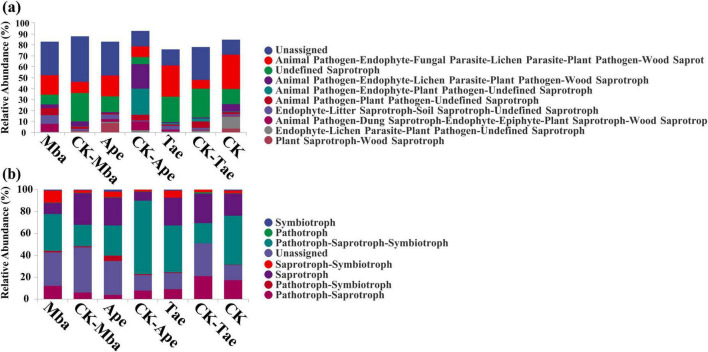
Relative abundance of fungal guild **(a)** and mode **(b)** in different samples predicted based on the FunGuild function.

On the basis of the ITS amplicon analysis, FunGuild can obtain the classification and abundance information of the fungal species present in the environment. Additionally, by classifying fungi, we can determine the ecological roles they play. As shown in Figure 5, we compared the 10 fungal functional groups with the highest relative abundances. The results revealed that, among all the samples, the most abundant were Unassigned, Animal Pathogen-Endophyte-Fungal Parasite-Lichen Parasite-Plant Pathogen-Wood Saprotroph and Undefined Saprotrophs. They accounted for 25.18, 17.85, and 17.02% of the dominant OTUs, respectively. In Ape, the abundances of Unassigned and Undefined Saprotroph guilds increased compared to those of the three plants growing in non-iron ore areas, whereas they decreased in Mba and Tae. Among the three plant species growing in the iron ore area, the abundances of the Endophyte-Litter Saprotroph-Soil Saprotroph-Undefined Saprotroph guilds increased, whereas the abundances of the Endophyte-Litter Parasite-Plant Pathogen-Undefined Saprotroph guilds decreased compared to those in non-iron ore areas.

**FIGURE 6:**
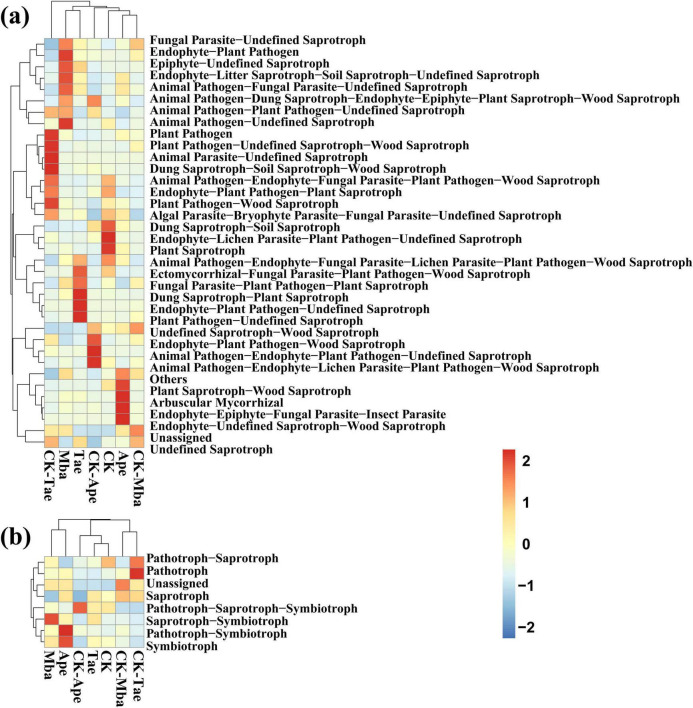
Heatmap of fungal guild **(a)** and mode **(b)** clustering for different samples based on FunGuild function prediction.

According to the annotated results of the database, the results of the functional abundance in Trophic Mode and Guild are counted, and the statistical results are shown in Figures 6a,b. Compared with those of CK-Mba, the values of Epiphyte-Undefined Saprotroph, Endophyte-Litter Saprotroph-Soil Saprotroph-Undefined Saprotroph, Animal Pathogen-Fungal Parasite-Undefined Saprotroph, and Animal Pathogen-Undefined Saprotroph modes were significantly greater (*P* < 0.05). The modes of the Plant Saprotroph-Wood Saprotroph, Arbuscular Mycorrhizal, and Entomophyte-Epiphyte-Fungal Parasite-Insect Parasitoid, and the Endophyte-Undefined Saprotroph-Wood Saprotroph decreased significantly in the Ape treatment compared to those in the CK-Ape treatment (*P* < 0.05). However, the modes of the Saprotroph, Animal Pathogen-Plant Pathogen-Undefined Saprotroph, and Animal Pathogen-Lichen Parasite-Plant Pathogen-Wood Saprotroph significantly increased (*P* < 0.05). In comparison to CK-Tae, Tae had significantly greater modes of Plant Pathogen-Wood Saprotroph, Plant Pathogen-Undefined Saprotroph-Wood Saprotroph, Animal Parasite-Undefined Saprotroph, and Stunted Saprotroph-Soil Saprotroph-Wood Saprotroph (*P* < 0.05), while the modes of Saprotroph-Plant Saprotroph, Endophyte-Plant Pathogen-Undefined Saprotroph, and Plant Pathogen-Undefined Saprotroph were significantly lower (*P* < 0.05). The dominant functional groups of the seven samples differed due to factors such as region, plant species, fertilization, and pesticides.

### 3.6 Correlation analysis of the fungal communities in rhizosphere soil and the soil physicochemical properties

**FIGURE 7:**
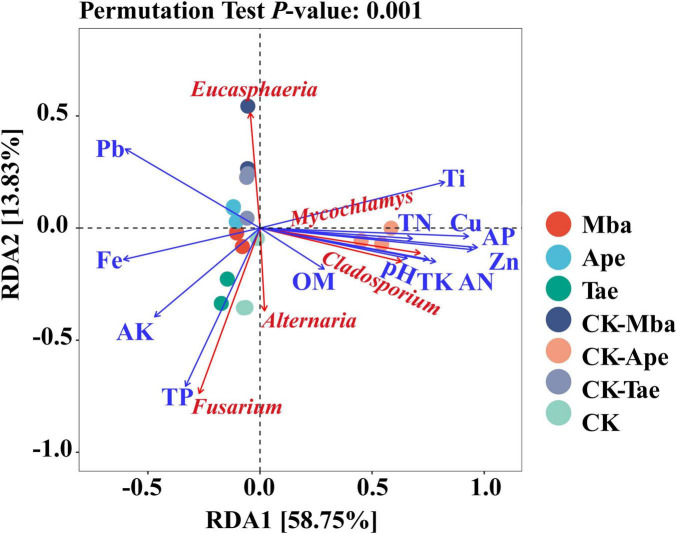
RDA analysis of rhizosphere soil fungi in relation to environmental factors.

The relationships between the relative abundances of the top five dominant fungal genera and the heavy metal concentrations and soil physicochemical properties in the rhizosphere soil were explored through RDA. Figure 7 illustrates that 67.58% of the variation in the microbial communities is explained by the first two axes of RDA. The microbial communities’ variation was explained by the first axis at 58.75%. pH, OM, AN, TK, TN, AP, Zn, Cu, and Ti were all positively correlated with it. And it was negatively correlated with TP, AK, Fe, and Pb. Cu had the smallest angle with the first axis and showed the largest correlation. The second axis accounts for 13.83% of the variation in microbial communities, with TP showing the least angle and correlation with this axis. The analysis also revealed that the dominant fungal genera in the soil were affected by multiple environmental factors, including positive correlations between *Mycochlamys* and *Cladosporium* and pH, OM, AN, TK, TN, AP, Zn, Cu, and Ti. *Eucasphaeria* showed a positive correlation with Pb only, which was not particularly strong. *Fusarium* had the greatest correlation with TP and was also affected by OM, AK and Fe. The physicochemical properties that were positively correlated with *Alternaria* were the greatest, including pH, OM, TN, TP, TK, AN, AP, AK, Zn, Cu, and Fe. The greatest correlation was with total phosphorus.

## 4 Discussion

The structure and diversity of soil fungal communities in the plant rhizosphere are mainly influenced by soil physical and chemical properties (Zhang et al., 2020; Su et al., 2022). The exploitation of mineral resources has led to the degradation of soil structure, which is reflected in the deterioration of the physical properties of the soil and the significant lack of chemical nutrients (Liu et al., 2023). In this study, it was found that the soil in the mining area was generally significantly weakly acidic. This change in physicochemical properties is closely related to the acid production from sulfide oxidation due to mining activities (Lu and Wang, 2012). At the same time, soil Fe, OM, and TK contents were significantly higher (*P* < 0.05). Among them, the enrichment of soil TK content may originate from the release of potassium ions due to mining disturbance (Jena, 2021), while the accumulation of soil OM content may be related to the inhibition of decomposing fungal activity by the acidic environment (Vylkova, 2017). Phosphorus showed a differential response, with elevated soil TP content and reduced AP content in Mba compared to CK-Mba. This may be the result of a combination of inorganic phosphorus release due to mining disturbance, as well as enhanced soil fixation and impeded microbial transformation (Short et al., 2000). Whereas, soil TP content was reduced but AP content was increased in Ape and Tae in mining compared to control, which may imply a reduction in phosphorus loss or release of fixed-state phosphorus (Grigore et al., 2023). Nitrogen cycling was also regulated by mining perturbations, with higher soil TN content but lower AN content in Mba compared to CK-Mba, which could be attributed to enhanced N fixation or changes in microbial activity (Liu et al., 2012). Quite the contrary, the soil TN content was reduced but AN content was elevated in Ape and Tae in mining compared to control, which may be related to accelerated organic N mineralization or reduced N loss (Mason-Jones et al., 2018). These results suggest that mining activities alter soil nutrient cycling through multiple pathways, which in turn affects the composition and function of fungal communities.

The composition and diversity of soil microbial communities in the plant rhizosphere are significantly regulated by soil environmental factors, a conclusion that has been extensively validated in multidimensional studies (Naz et al., 2022; Zeng et al., 2024; Wang et al., 2007; Narendrula-Kotha and Nkongolo, 2017). By comparing the structural characteristics of plant rhizosphere microbial communities in different habitats such as tailings topsoil, mining cave topsoil, open pit topsoil, and deep open pit soil, previous studies (Qiu et al., 2024) have found significant differences in rhizosphere soil microbial community structure and diversity among plant species. The present study further confirms that iron ore mining activities have a profound effect on the Alpha and Beta diversity of rhizosphere fungal communities by altering soil physicochemical properties (e.g., heavy metal Fe enrichment, pH acidification) and plant-microbe interactions patterns. Specifically, Simpson, Shannon, and Pielou evenness indices of fungal communities in the rhizosphere soils of the three plant species (Mba, Ape, and Tae) were generally lower in the mining area than in the control, whereas Dominance was generally elevated, indicating that the mining activities reduced the diversity and homogeneity of the fungal communities. It is worth noting that although the species richness indicators (Chao1 index and number of observed OTUs) of the Tae rhizosphere showed a non-significant upward trend, the decrease in its Pielou evenness still suggests that the over proliferation of dominant species (e.g., Fusarium) in the mining environment creates an ecological niche squeeze on other species. Notably, there were differences in the response of different plant taxa to mining disturbance. Only the Shannon index showed a significant decrease (*P* < 0.05) in the Mba group, indicating that the species richness of its rhizosphere fungal community was strongly influenced by mining, but the change in community evenness was not significant. The Simpson index, Dominance index, and Pielou evenness index of the Ape group were significantly changed (*P* < 0.05). This may be related to its high dependence on mycorrhizal symbiosis, resulting in a more sensitive rhizosphere fungal community to changes in the soil environment (Bonfante and Genre, 2010). All four diversity indices of the Tae group showed significant differences (*P* < 0.05), likely due to their shallow root structure that makes them more exposed to surface-contaminated soil. And this exposure leads to an imbalance in microbial homeostasis (Grose et al., 1996). Based on Beta diversity analysis (PCoA, NMDS), this study further reveals that there are differences in the rhizosphere soil fungal community structure of the three plant species in mining and control areas. This finding is in line with the results of previous studies on the impact of mining activities on microbial communities (Liu et al., 2019). Notably, iron ore mining reduced the variability of fungal communities among different plants, suggesting that mining activities may have weakened plant-specific regulation of rhizosphere microbial communities by homogenizing the soil environment (Tedersoo et al., 2014). Overall, iron ore mining affected the diversity of rhizosphere soil fungi, while the magnitude of the effect was related to plant species.

Long-term mining activities have caused considerable alterations to the diversity of soil fungal communities in the plant rhizosphere, resulting in significant changes in fungal abundance and community structure (Li et al., 2022; Chen et al., 2020b). At the genus level, the main dominant fungal taxa include *Fusarium*, *Mortierella*, *Mycochlamys*, and *Cladosporium*. Among them, the relative abundance of *Fusarium* was generally higher in the mining area than in the control area, which may be related to its tolerance mechanisms (e.g., metal chelation, efflux pumping, etc.) to heavy metals (e.g., Fe) (El-Sayed et al., 2022). However, the enrichment of *Fusarium* as a potential plant pathogen may increase the risk of plant disease (El-Sayed et al., 2022). *Mortierella* was more abundant in the rhizosphere soil of all three plant species in the mining area than in the control area. This genus of fungi is not only involved in the decomposition of organic matter and nutrient cycling, but also synthesizes a variety of bioactive substances (e.g., surfactants, antibiotics, etc.), which may contribute to the adaptability of plants in polluted environments (Cui et al., 2017). The distribution pattern of *Mycochlamys* varied according to plant species, with its abundance being significantly higher in Mba than in CK-Mba, and lower in Ape and Tae than in the control. The function of *Mycochlamys* may vary depending on the host plant (Wang H. et al., 2023; Liu Y. et al., 2024). In contrast, *Cladosporium* was less abundant in the rhizosphere soils of all three plant species in the mining area than in the control area. This is consistent with the results of existing studies. This study indicated that the abundance of several fungi, including *Cladosporium*, was significantly reduced in moderately and highly contaminated soils (Xu et al., 2023). These results suggest that mining activities and plant species together regulate the composition and abundance of soil fungal communities in the rhizosphere, which may affect the functional stability of the ecosystem.

The relative abundance table and heatmap of the FUNGuild function prediction show that the guilds and modes of fungi increase during iron ore mining activities. For example, the Animal Pathogen-Endophyte-Fungal Parasite-Lichen Parasite-Plant Pathogen-Wood Saprotroph and Endophyte-Litter Saprotroph-Soil Saprotroph-Undefined Saprotroph guilds as well as the Symbiotroph, Saprotroph-Symbiotroph, and Pathotroph-Symbiotroph modes. The guilds and modes of fungal communities vary according to species, environment and nutritional mode (Ma et al., 2022). In this study, researchers found that fungi adapted to stressful conditions by enriching specific guilds and modes in an iron ore mining environment.

## 5 Conclusion

This study systematically investigated the effects of iron ore mining activities on the structure and function of soil fungal communities in the rhizosphere of *Musa basjoo*, *Apegdalus persica*, and *Triticum aestivum* L. The results showed that the synergistic effect of soil acidification and heavy metal Fe enrichment significantly inhibited the survival of sensitive taxa such as *Cladosporiaceae* and *Sarocladium*, resulting in an overall decreasing trend in Alpha diversity indices (Shannon index, Simpson index, and Pielou evenness). Functional prediction analyses showed that the Animal Pathogen-Endophyte-Fungal Parasite-Lichen Parasite-Plant Pathogen-Wood Saprotroph guild dominated the plant rhizosphere soils in the mining area. Redundancy analysis (RDA) revealed that soil property parameters (including pH, iron content, and total phosphorus content) showed a strong correlation with fungal community composition. This further confirmed that the alteration of soil physicochemical properties caused by mining activities is a key factor driving changes in microbial community structure. This study systematically reveals the differential effects of iron ore mining on soil microbial communities in the rhizosphere of different plants and elucidates the response strategies of major fungal taxa to mining perturbations. Based on the results of the study, it is suggested that future research needs to focus on the mechanisms of key fungal taxa in heavy metal tolerance. This should be done by combining multi-omics analysis, studying plant-fungal interaction mechanisms, and conducting long-term field experiments. These efforts will provide a theoretical basis and technical support for the ecological remediation of soil in mining areas.

## Data Availability

The original contributions presented in the study are included in the article/Supplementary material, further inquiries can be directed to the corresponding authors.
