# Supplementary figures and images for: Impact of iron ore mining activities on the rhizosphere fungal communities of Musa basjoo, Apegdalus persica, and Triticum aestivum L
Source: Front Microbiol. 2025 Jun 27;16:1592479. doi: 10.3389/fmicb.2025.1592479 (PMC12246942; doi:10.3389/fmicb.2025.1592479)

Figure S1. Dilution curves of plant rhizosphere soil fungal communities

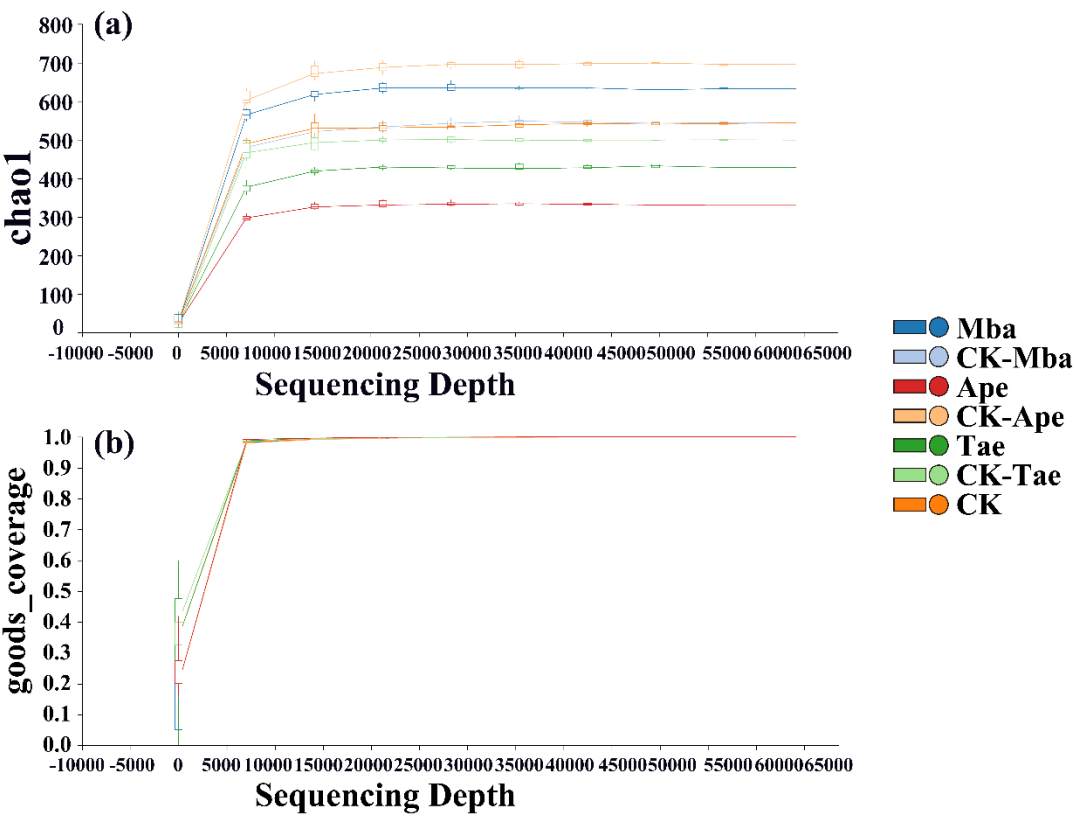

Supplement: Supplementary file 1 [file Data_Sheet_1.pdf]
